# High Sensitive Night-time Light Imaging Camera Design and In-orbit Test of Luojia1-01 Satellite

**DOI:** 10.3390/s19040797

**Published:** 2019-02-15

**Authors:** Zhiqiang Su, Xing Zhong, Guo Zhang, Yanjie Li, Xiaojun He, Qiang Wang, Zongxi Wei, Chunling He, Deren Li

**Affiliations:** 1 Chang Guang Satellite Technology Co. LTD, Changchun 130102, China; suzhiqiang927@163.com (Z.S.); liyanjiework@126.com (Y.L.); hexiaojun6@163.com (X.H.); wangqiang200929@126.com (Q.W.); weizongxi@charmingglobe.com (Z.W.); hechunling14@163.com (C.H.); 2 Changchun Institute of Optics, Fine Mechanics and Physics, Chinese Academy of Sciences, Changchun 130033, China; 3 State Key Laboratory of Information Engineering in Surveying, Mapping and Remote Sensing, Wuhan University, Wuhan 430079, China; guozhang@whu.edu.cn (G.Z.); drli@whu.edu.cn (D.L.)

**Keywords:** Luojia1-01 satellite, night-time light camera, remote sensing, signal to noise ratio, special shaped hood

## Abstract

Luojia1-01 satellite is the first scientific experimental satellite applied for night-time light remote sensing data acquisition, and the payload is an optical camera with high sensitivity, high radiation measurement accuracy and stable elements of interior orientation. At the same time, a special shaped hood is designed, which significantly improved the ability of the camera to suppress stray light. Camera electronics adopts the integrated design of focal plane and imaging processing, which greatly reduces the volume and weight of the system. In this paper, the design of the optical camera is summarized, and the results of in-orbit imaging performance tests are analyzed. The results show that the dynamic modulation transfer function (MTF) of the camera is better than 0.17, and the SNR is better than 35 dB under the condition of 10 lx illuminance and 0.3 reflectivity and all indicators meet the design requirements. The data obtained have been widely applied in many fields such as the process of urbanization, light pollution analysis, marine fisheries detection and military.

## 1. Introduction

Night-time light remote sensing image refers to the night-time image of the earth obtained by the optical remote sensing device. Currently the world’s two major night-time light sensors are the United States defense meteorological satellite program (DMSP) with a linear scanning business system (OLS) and polar environment business satellite (S-NPP) carrying the visible infrared imaging radiometer (VIIRS) [1,2,3,4], and their data have been used in gross domestic product, population, carbon emissions, armed conflicts, power consumption and mapping urban extents [5,6,7,8,9,10,11]. The quality of remote sensing data acquired is mainly determined by spatial resolution radiation measurement accuracy, dynamic range and sensitivity. OLS and VIIRS have spatial resolution of 2.7 km and 740 m and dynamic range of 6 bit and 14 bit respectively. Jilin-1 video satellite developed by Chang Guang Satellite Technology Co., Ltd. (Changchun, China) can provide 1 m resolution night-time light color remote sensing images and is the highest spatial resolution satellite used in this field by far. Considering the spatial resolution, width and the influence of radiation measurement precision’s comprehensive effect on remote sensing data, Wuhan University developed Luojia1-01 satellite, which has been successfully launched on 2 June 2018 [12,13,14,15]. The main payload is a camera which can obtain nightlight images of the earth. The camera innovatively uses the combination of a large relative aperture optical system and high sensitivity detector to improve the signal to noise ratio (SNR) of dark scenes. and the spatial resolution is 130 m with 12 bit dynamic range. In addition, we designed a special hood to effectively avoid the influence of solar radiation on camera imaging. In this paper, the design and in-orbit test of night light camera is introduced. Results of in-orbit test show that all the specifications of the camera meet the requirements, and that the camera has shown a good performance.

## 2. Mission analysis

### 2.1. Features of Night Light

The visible band radiation information of night-time obtained by optical remote sensing devices mainly comes from human activity light, among which the light on land is mainly urban light, and the light on sea surface mainly comes from fishing boat light and drilling platform [12]. In accordance with the requirements of Chinese urban road lighting, the main and trunk roads of the city generally use high pressure sodium lamp as the light source [13], the spectral distribution of which is concentrated in the spectrum range of 500 nm to 900 nm.

Nightlight source has a low illuminance. According to Chinese urban road lighting standards, the illuminance maintenance value of fast road and main road is 20 lx to 30 lx, and the secondary road is 10 lx to 15 lx, while branch road is just 8 lx to 10 lx, which are far less than the illuminance values of ground during the day [16]. When the moon acts as the light source, the maximum illuminance is about 0.2 lx [17].

When imaging a low-illuminance target, the signal-to-noise ratio (SNR) of the space camera becomes the main factor restricting the imaging quality. In order to improve the SNR of camera imaging, photodetector with high sensitivity and low noise is needed, and optical imaging system with large relative aperture is required.

### 2.2. Identification of Imaging Methods

The main imaging methods of space camera include line-array push-broom imaging, plane-array gaze imaging, and frame push-broom imaging. Linear array push-broom imaging has strict requirements on satellite’s attitude control, stability and pointing accuracy, and it is easy to produce image shift and imaging quality will be reduced. Due to the absence of image shift mismatch in the plane-array gaze imaging mode, images with high SNR can be obtained by long-time integration, but the imaging width is limited. Frame push-broom can obtain the images with larger width by changing the frame frequency of the camera, the overlap rate of adjacent frames of images can be controlled, which is of great significance for multi-temporal images matching, dynamic targets monitoring, etc. Meanwhile, the frame push-broom mode requires relatively low attitude control of the satellite, so we choose frame push-broom imaging mode as Luojia1-01 night-time light camera’s main imaging mode [18,19].

### 2.3. Requirements for Payload

The main evaluation indexes of space camera include SNR and MTF (modulation transfer function) of the images. SNR reflects the radiation resolution for visible light camera, which is reflected in the cleanliness of the image and SNR is of great significance for target classification. MTF reflects the frequency characteristics of information transmitted by the photoelectric imaging system, which depends on the diffraction and aberration of the optical system and the characteristics of the photoelectric sensor. MTF reflects the sharpness of image and can evaluate image quality comprehensively. Because the luminous intensity of ground targets is very low, it is essential to obtain image data with high SNR. According to the requirements of radiation quality, SNR is required to reach 20 dB under the condition of ground illuminance of 10 lx and reflectance of 0.3. Static MTF is required to be better than 0.2 at the Nyquist frequency of the camera.

## 3. Optical and Mechanism Design

### 3.1. Decomposition for System Indicators

Imaging performance of the space camera is mainly determined by optical imaging system and photodetector. Photoelectric conversion ability and noise level of photodetector have important influences on the camera’s capability.

According to the requirements of the task, a CMOS (Complementary Metal Oxide Semiconductor) detector with 2048 × 2048 pixels manufactured by GPIXEL Inc. is selected. The pixel size of the detector is 11 microns, and the main parameters of the photodetector are shown as Table 1.

**Figure 1:**
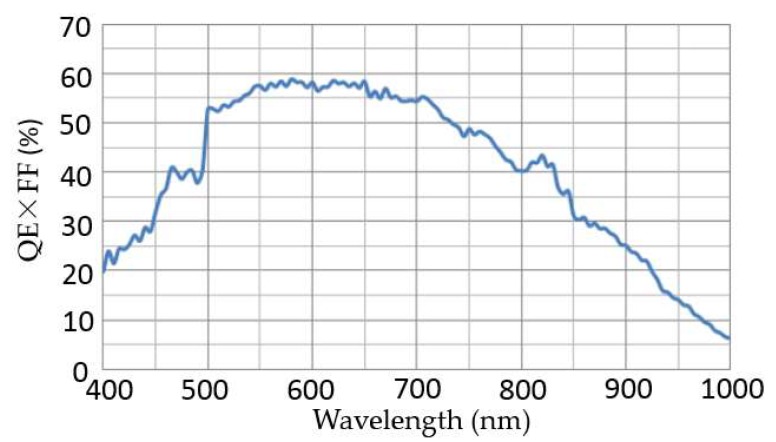
Spectral response curve for the detector.

Spectral response is an important parameter of the detector, which determines the photoelectric conversion capability of the system. The spectral response curve of the detector is shown in Figure 1.

The input parameters for optical system design mainly include optical system focal length, field of view and relative aperture.

Ground sample distance (GSD) of space camera is related to the satellite orbit height H, pixel size (a) and focal length (f) of the optical system.

The orbit height is 645 km, so the focal length of the optical system is required to be 55 mm in order to achieve the spatial resolution of 130 m at the nadir point.

At this time, the imaging width of the camera is 264 km, which meets the requirement that the width is larger than 200 km.

Large relative aperture optical system has the advantages of high energy collection capacity and less affected by diffraction, so it is beneficial to improve the imaging SNR and MTF. But, the volume and weight of the system will increase rapidly, and the aberrations of the system are not easy to correct. The influence of F number (reciprocal of the relative aperture) on system performance is analyzed below.

**Figure 2:**
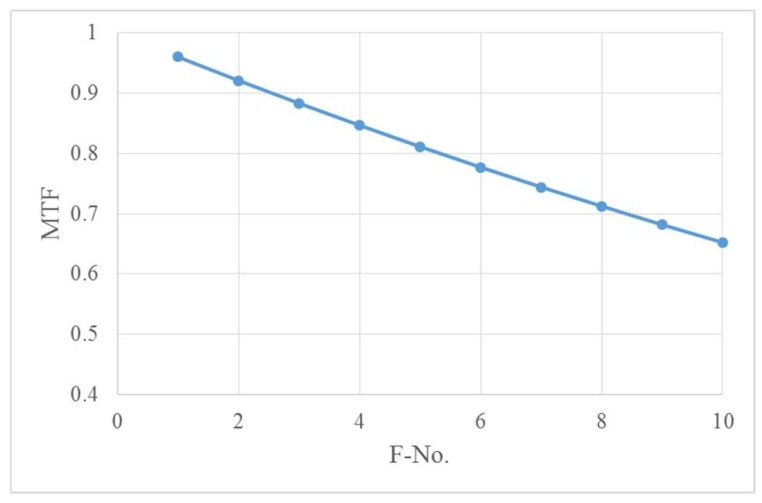
The relationship between MTF (modulation transfer function) limited by diffraction and F number.

Since the camera’s static MTF is targeted to be greater than 0.2, the optical system transfer function after adjustment is required to be greater than 0.5 considering the detector’s MTF. The relationship between MTF and F number at Nyquist frequency (46 lp/mm) is calculated, which is shown in Figure 2.

**Figure 3:**
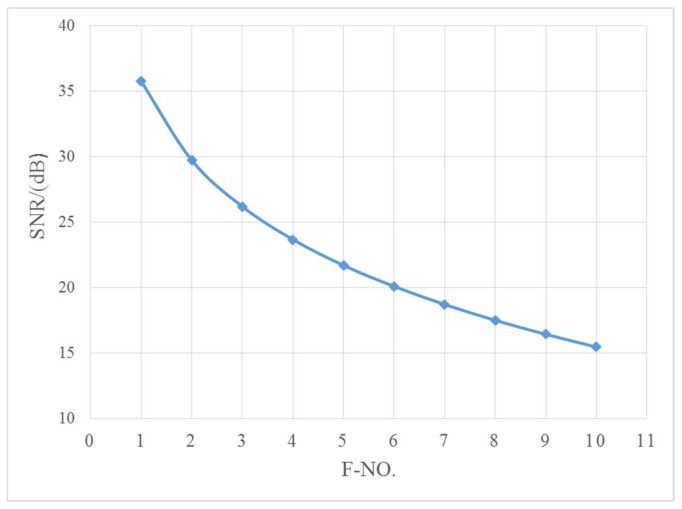
Relationship between SNR(signal to noise ratio) and F-No. of optical system.

In addition, the number of signal electrons produced by the camera is inversely proportional to the square of the F number of the optical system. As required by the task, the imaging SNR of the system is estimated. Among which, atmospheric transmittance is 0.6, optical system transmittance is 0.7 and target reflectivity is 0.3 with 10 lx illuminance, and the target is assumed to be a lambert body. Then, the relative aperture of the optical system is an important parameter determining SNR, The influence of F number on SNR of the photoelectric imaging system is analyzed, which is shown in Figure 3.

Considering comprehensively the influence of the MTF, SNR and aberration correction, the design is balanced and the F number of the optical system is set to 2.8 which satisfies the requirements of the system for SNR and MTF.

### 3.2. Optical System Design

**Figure 4:**
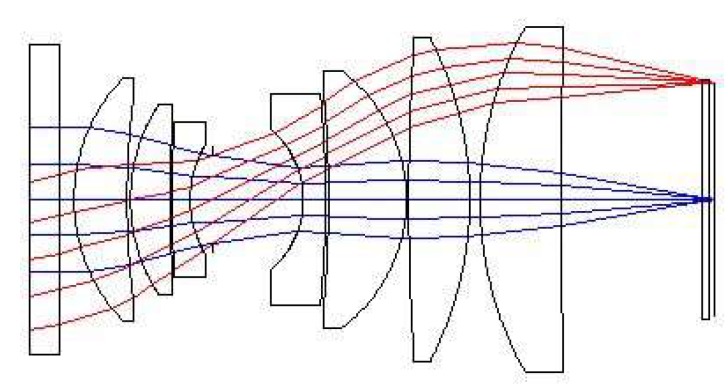
The structure of the optical system.

According to the decomposition results of the above system indexes, this system is designed by refraction optical system, which has medium field of view and large relative aperture. According to the requirements of field of view and relative aperture, the optical system in the form of double Gaussian is selected for optimal design. The distortion of the system should be controlled in the design process, and the requirements of image quadric centroid should be satisfied. The design results are shown in Figure 4.

**Figure 5:**
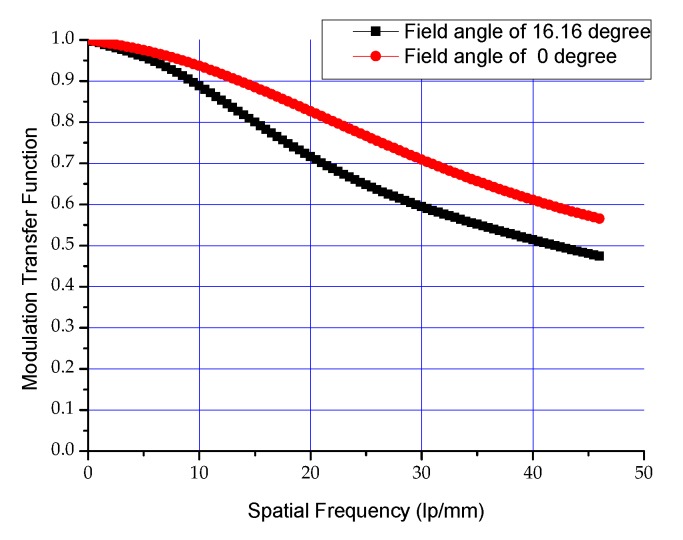
Modulation transfer function curves of the optical system.

The designed system meets the requirements of image quadric centroid, and the modulation transfer function curve of the system is shown in Figure 5.

Optical system’s MTF value at Nyquist frequency is higher than 0.5 and this can ensure the camera’s static MTF is better than 0.2.

**Figure 6:**
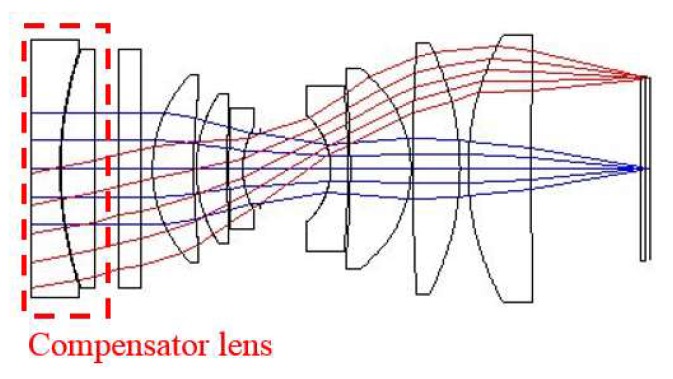
Optical system’s structure with compensation lens.

Given that the camera works in a vacuum environment in orbit but the assembly and test are completed under normal pressure, a compensation lens is designed to compensate for the influence of air pressure on the imaging quality of the optical system. The function of the compensation lens is to ensure that the back intercept of the optical system with adjustment lens under normal pressure is the same as that without compensation lens in vacuum. The designed compensation lens is composed of two pieces of transmission elements, and the material is H-K9L. The system structure with the compensation lens is shown as Figure 6.

After the assembly of the camera, MTF of the camera was tested by the slant edge method. The test results show that the MTF of the camera is 0.247 at Nyquist frequency [20].

**Figure 7:**
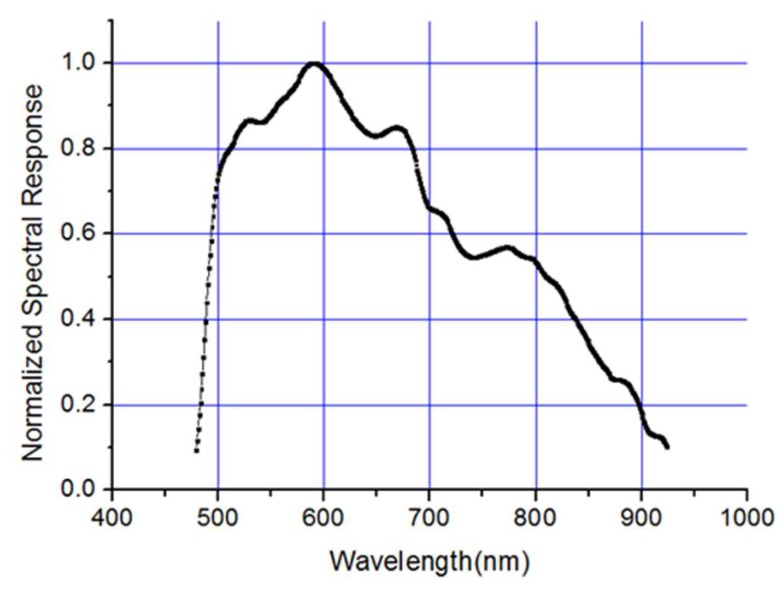
The relationship between MTF limited by diffraction and F number.

In addition, we also tested the spectral response of the system, and obtained the normalized spectral response curve of Luojia1-01 satellite by means of mono-chromator scanning, as shown in Figure 7.

### 3.3. Mechanical Structure Design

**Figure 8:**
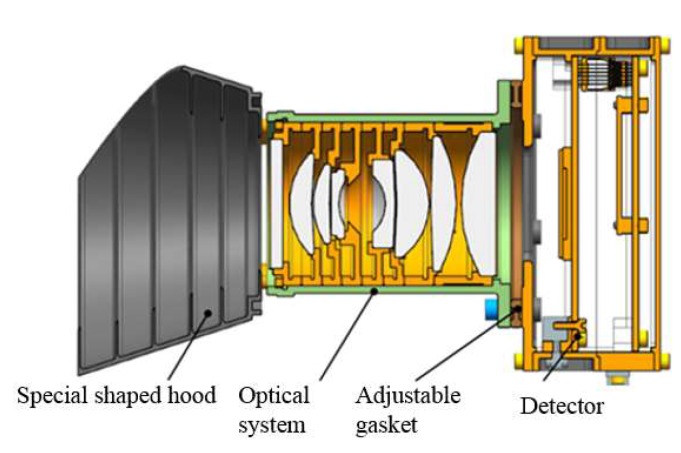
Mechanical structure of the camera.

Luojia1-01 camera is mainly composed of camera lens, focal plane electric box and hood. The lens is installed in the lens seat by rolling edge method and assembled according to the tolerance requirements of optical design. The lens is connected to the focal plane electric box by screws and the focal plane position is adjusted by a gasket to ensure all the regions of the detector can get good performance. The designed hood is located at the front of the lens. The system profile is shown as Figure 8.

In order to ensure the stability of the optical system, titanium alloy is selected as the structural design material. Its advantage is that the thermal expansion coefficient is close to the optical material selected. The design of camera structure mainly takes into account the assembly tolerance requirements of optical system, the mechanical characteristics and thermal characteristics of the system [21].

## 4. Design and Analysis of StrayLight Elimination

### 4.1. The Effect of Stray Light on Imaging

In imaging optical system, some non-imaging light can reach the focal plane and become stray light. Stray light can reduce the imaging SNR and MTF. In particular, when imaging low-illuminance targets, stray light may drown the signal and make the system disabled. Therefore, it is necessary to analyze and suppress the stray light [22,23,24].

Due to that the scattering radiation of the atmosphere is small at night, its influence on imaging can be ignored. But when the satellite observes shadow region, especially near the terminator, solar radiation may be scattered into the optical system by the camera hood or other parts of the satellite. Since the solar radiation energy is much higher than the emission or reflection energy of ground targets, it will be disastrous for imaging. Response of the sensor caused by object targets is related to the optical system’s relative aperture, and response caused by solar radiation is relative to optical system’s PST (Point spread transmittance).

The irradiance of the focal plane generated by the ground target can be calculated according to the following equation:

(1) $$E_{1}=\frac{\rho E\tau_{a}\tau_{o}}{4F^{2}}$$

Parameters in formula 1 are defined as Table 2.

The irradiance of the focal plane generated by the solar radiation can be calculated according to the following formula:

(2) $$E_{2}=E_{sun}\times PST(\theta )$$

where $E_{sun}$ denotes the irradiance of the sun at the camera. According to Planck’s law, the solar illuminance is calculated to be 608 $W/m^{2}$ within the working spectrum range of the optical system.

PST(θ) denotes the PST value at different angles of incidence.

Based on the discussions above, we can see that for a target with 1 lx illuminance and 0.3 reflectivity, and the transmittance of atmosphere 0.6, when PST is $10^{-8}$, irradiance on the focal plane caused by object and solar is on the same level. We wish the system’s PST to be less than $10^{-10}$ to protect the system from solar radiation.

### 4.2. Analysis of the Relationship Between Solar Vector and Camera Attitude

**Figure 9:**
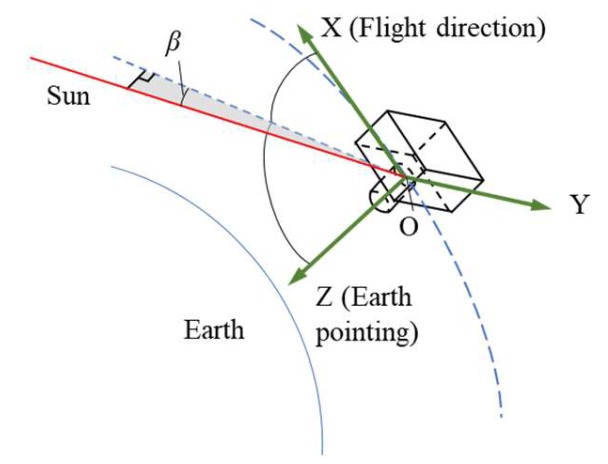
The relationship between solar vector and the camera coordinate system.

**Figure 10:**
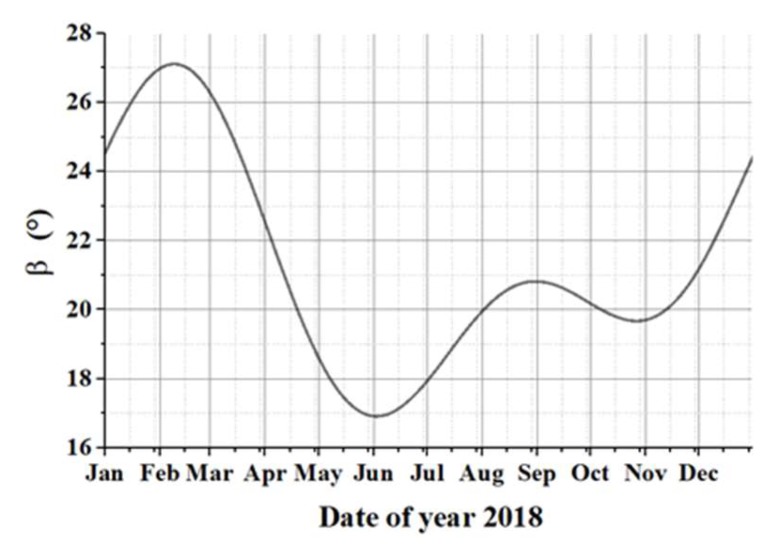
The changes of the angle between the solar vector and orbital plane changes in one year.

The relationship between the sun vector and the camera optical axis can be represented by the azimuth and altitude angle of the sun vector in the camera coordinate system and the meaning of different angles are shown as Figure 9. Simulation is carried out through STK software, [25] we can see that the sun vector in orbital plane angle beta ranges from 17 degree to 27degree, which is shown as Figure 10.

**Figure 11:**
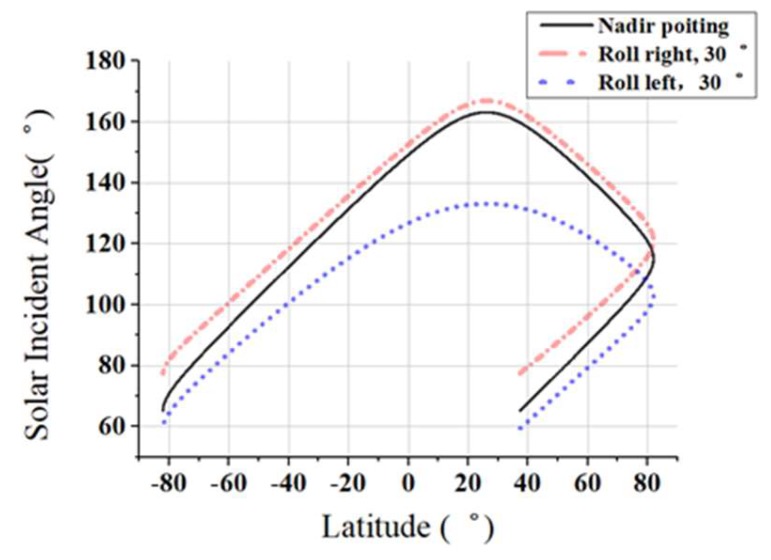
The angle between solar vector and camera axis.

Angle is smallest on June 3rd of each year, therefore, the relative position of the satellite and the sun is analyzed on this day to obtain the minimum angle between the sun vector and the camera axis. As the satellite has the ability to observe the earth with a swing angle of ±30°, we analysis three different kinds of conditions: without swing, rolling right 30° and rolling left 30° and results are shown as Figure 11. The minimum angle between solar vector and the camera optical axis is 52°.

### 4.3. Special Shaped Hood Design

**Figure 12:**
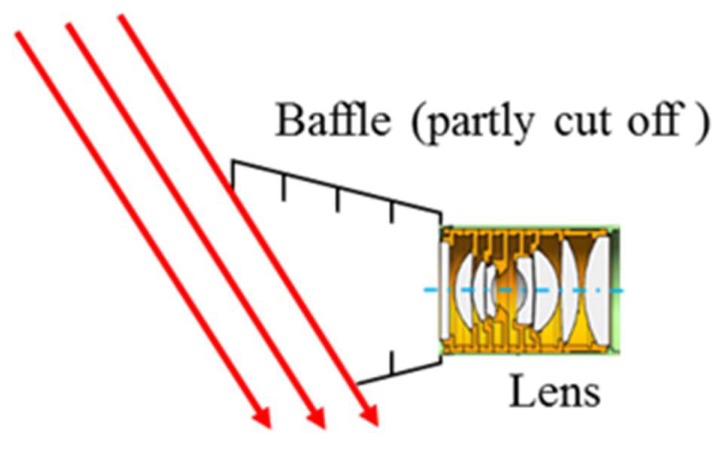
Profile of the camera’s special shaped hood.

Considering the influence of solar radiation on camera imaging, a traditional hood cannot meet the requirements of imaging, therefore, a special shaped hood is designed. The objective of the special- shaped hood design is to prevent solar radiation from scattering through the structural parts into optical system. The designed hood is shown as Figure 12.

On the basis of the traditional hood, and considering the influence of the size of the satellite, the special-shaped hood is cut with the azimuth angle 22 degree and 52 degree with optical axis. When the angle between the solar vector and the camera axis is greater than 52 degree, the solar vector cannot enter the optical system through the hood, and the camera will not be affected by solar radiation.

**Figure 13:**
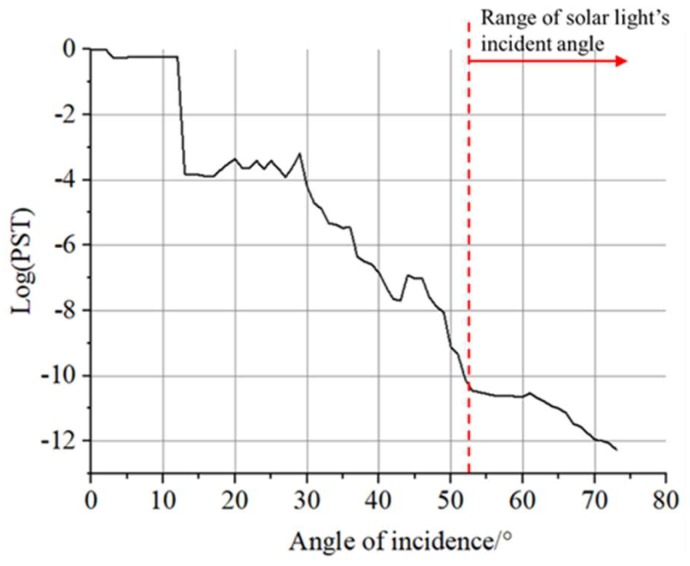
PST curves of the camera.

Stray light analysis on satellite model is necessary because other components of the satellite can also reflect or scatter solar radiation and become stray light for camera imaging. The model is analyzed in the optical analysis software Tracepro and the PST of the camera is calculated [26]. The calculation results are shown as Figure 13.

As can be seen from the figure above, when the angle between the incident solar vector and the camera optical axis is greater than 52 degree, the PST of the camera decreases rapidly, and the influence of solar radiation on the camera can be ignored.

### 4.4. In-orbit Verification of the Hood

**Figure 14:**
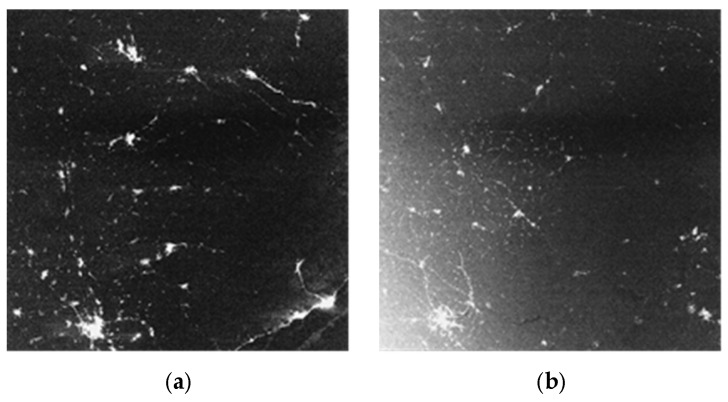
Different frames of night-time light image of Moscow area in one orbit. (**a**) normal image with the angle between axis and solar vector greater than 52 degree; (**b**) image effected by stray light with the angle between axis and solar vector less than 52 degree.

In order to verify the performance of the designed hood, a push-broom imaging for the Moscow area was carried out at 3:09 on June 21th, 2018 and the image is shown in Figure 10. The angle between optical axis and solar vector has been changing during the whole process and when it decreased to 52 degree, solar radiation was scattered into the optical imaging system and became stray light. As can be seen from Figure 14a, images are not effected at the beginning, and the angle between solar vector and camera axis is larger than 52 degree, when the angle decreases to 52 degree, stray light appears and images are seriously effected, which is shown in Figure 14b. Both the two images are uncropped and are whole frame with swath 260 km. In-orbit imaging data show that the design of the special-shaped hood has an obvious effect on restraining solar radiation and can effectively improve the operational efficiency of the satellite.

## 5. HDR mode design

### 5.1. Imaging Electronics Design

Luojia1-01 night-time light camera adopts the design of integrating imaging, compression, storage and other functions through combining the imaging board and data processing board together. This design method can effectively reduce the size and weight of the camera, which is of great significance for the application of micro-satellite platform.

The control center of the camera is FPGA (Field-Programmable Gate Array). Under the control of FPGA, image data reception and forwarding, configuration and data flow management of ADV212, solid-state disk storage management and external communication management of the whole system are realized.

### 5.2. High Dynamic Range Image Construction

The radiance of different targets at night are quite different, which requires the camera to have a high dynamic range to obtain more information of the scene. In order to improve the dynamic range of the camera, we designed the HDR mode. The method adopted was that all the pixels could output the gray value of different gains in one imaging, that is, the high gain image and low gain image of the scene could be obtained simultaneously. The high and low gain image output by the camera can be reconstructed to obtain the image with a high dynamic range. According to the characteristics of the camera, when the output value of the high gain image is less than the threshold Tn, it is considered to be in the linear region of the sensor, and the high gain output is considered to be effective. When the DN value of the high gain image is greater than Tn, the high gain image data is invalid. The corresponding low gain image output value is used for conversion. The specific implementation method is as follows:

(3) $$Y_{HDR}=\left\{ \begin{array}{l} Y_{H}(Y_{H}\leq T_{H})\\ aY_{L}-b(Y_{H}\geq T_{H}) \end{array}\right.$$

The parameters and their meanings are listed in Table 3.

**Figure 15:**
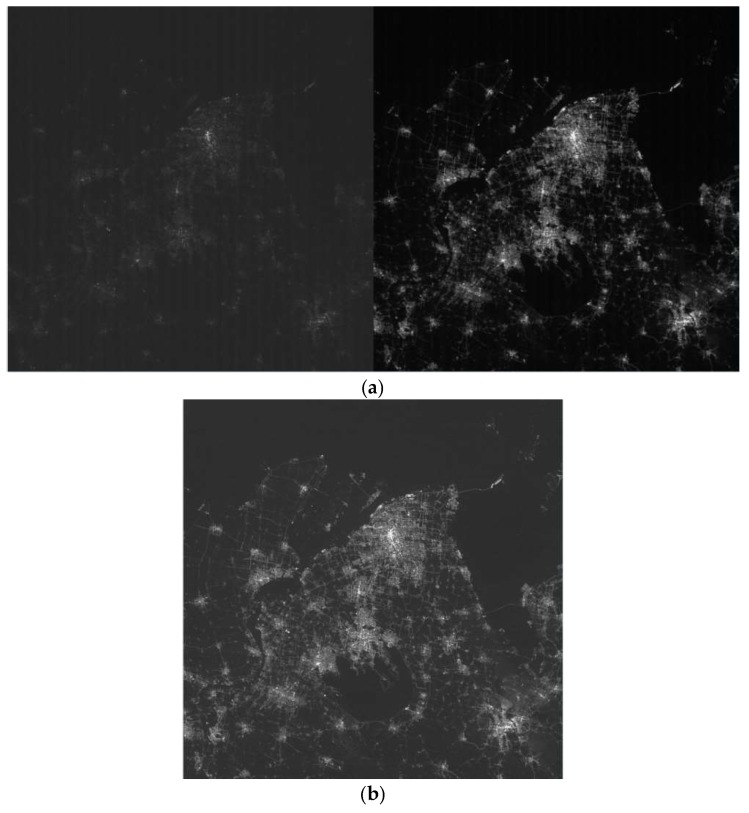
(**a**) Original image of low gain and high gain mode; (**b**) HDR image constructed by low gain and high gain image.

The parameter $T_{H}$ we selected is 3800 DN according to the response for high gain image. Figure 15 shows the original image and the high dynamic range image of Shanghai city in China.

## 6. In-orbit test results

### 6.1. Dynamic MTF Evaluation in orbit

The main methods for measuring the in-orbit MTF of remote sensing images are point pulse method, line pulse method and slant edge method [27,28,29,30]. According to the imaging characteristics of Luojia1-01 nightlight camera, MTF is calculated by using the line diffusion function based on line targets.

**Figure 16:**
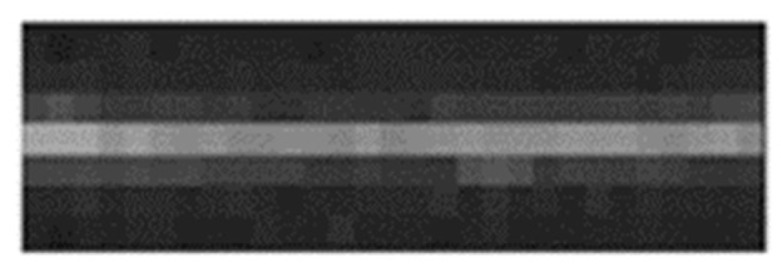
Line target in an image.

We select a long-straight bridge target in the image which is shown as Figure 16 to calculate MTF. The image is interpolated and the line spread function (LSF) can be further extracted. Then MTF is obtained by differentiating the line spread function.

**Figure 17:**
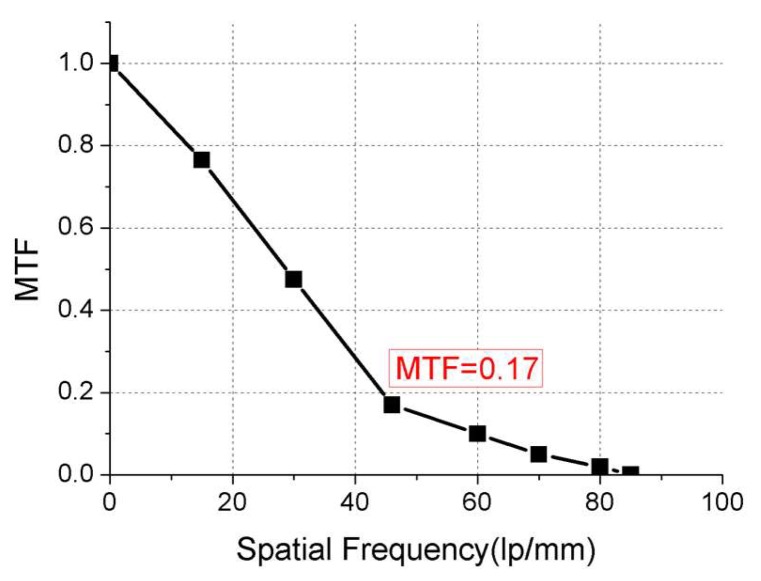
Dynamic modulation transfer function curves of Luojia1-01 satellite.

According to Figure 17, the camera’s dynamic MTF at Nyquist frequency (46 lp/mm) is 0.17, which meets the requirement that dynamic MTF should be better than 0.15.

### 6.2. SNR evaluation of the camera in-orbit

Traditional signal-to-noise ratio (SNR) test based on image requires that there is an uniform region [31]. However, this is not easy to achieve in night-time images. We proposed a new SNR calculation method using time series images. Detailed methods are as follows:

(1) Adjust the frame frequency of the camera, and then continuous multi-frame images can be obtained for the same ground area in one push-broom imaging.

(2) Through image registration, multiple exposure images of the same target point are obtained. Signal and noise can be calculated respectively on the time series and SNR can be obtained with unit of dB through formula 4.

(4) $$SNR=20\times \log_{10}^{\frac{Signal}{Noise}}$$

(3) According to the absolute radiation calibration data, we will get the relation curve between SNR and radiance. We can obtain the relationship, which is between radiance of the camera’s entrance pupil and illuminance on the objects, based on the assumption that the objects observed are lambert body with reflectivity of ρ and the atmosphere’s transmittance τ. The equation is shown below:

(5) $$L=\frac{E\times \rho \times \tau }{\pi \times K_{\lambda }}$$

**Figure 18:**
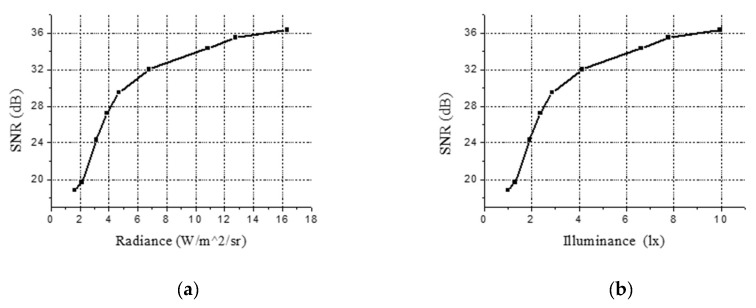
SNR(signal to noise ratio) tested results of the images. (**a**) the relationship between SNR and radiance of optical entrance; (**b**) the relationship between SNR and illuminance of targets with reflectivity of 0.3 and atmosphere’s transmittance of 0.6.

Here, $K_{\lambda }$ represents the visual function, which is related to the spectral distribution of the light source. According to the spectrum of the sodium vapor lamp, test results are shown in Figure 18.

It can be seen that under the condition of 10 lx illuminance, when the object reflectivity is 0.3 and the atmospheric transmittance is 0.6, the image SNR obtained can reach 35.5 dB, completely meeting the requirements of the design task and effectively ensuring the radiation measurement accuracy of the system.

## 7. Conclusions

Through the design and development of Luojia1-01 satellite’s payload, the research of high sensitivity optical remote sensing camera for micro-nano satellite platform is explored. The in-orbit test results show that the performance of the camera meets all the design requirements.

Innovation of Luojia1-01 satellite nighttime light camera are shown in the following aspects:

(1) The combination of large relative aperture optical system and large pixel high sensitivity CMOS detector is adopted, and the imaging mode of frame push-broom is adopted to ensure the radiation sensitivity and geometric stability of the camera.

(2) Fully considering the characteristics of the satellite in-orbit imaging environment, in view of the traditional hood’s disadvantage that cannot suppress solar radiation, we designed a special- shaped hood. In-orbit test results show that the design of hood effectively reduces the influence of solar radiation on imaging and test results are consistent with theoretical analysis. The design of the special shape hood is of great significance for micro satellite platforms.

(3) The adoption of HDR imaging mode greatly improves the dynamic range of im aging and enables accurate measurement of different radiance targets in the scene. Meanwhile, the integrated design of electronics is of great significance for the optical micro-satellite platform.

As a professional nightlight remote sensing satellite, Luojia1-01 has acquired a large number of remote sensing data and completed the drawing of a global map. The data obtained has been widely used in many fields.

However, current high-sensitivity nightlight remote sensing cameras can only obtain grayscale images, while spectral information of targets is also quite important, so we believe that multi-spectral information with high SNR nightlight optical camera and other methods such as polarization remote sensing will be the future direction in nightlight remote sensing field.

## Figures and Tables

**Table 1:** Parameters of the CMOS detector.

Parameters	Value
Active area	22.5 mm (H) × 22.5 mm (V)
Pixel size	11 μm × 11 μm
Number of active detectors	2048 × 2048
Full well/Ke-	91
Readout noise (e-)	1.47
Dark current (e-/s/pix) @ 25 °C	32
Dynamic range (Standard mode)	>70 dB
Dynamic range (High dynamic range mode)	>96 dB
Working temperature (°C)	−55-+80
Power consumption (mW)	<600
ADC	12

**Table 2:** Physical meaning of parameters in formula 1.

Parameter	Physical Meaning
ρ	Reflectivity of ground object
E	Illuminance of ground object
$\tau_{a}$	Transmittance of atmosphere
$\tau_{o}$	Transmittance of optical system
F	Reciprocal of the relative aperture

**Table 3:** Parameters and physical meanings in formula 3.

Item	Explanations
Gain ratio	$a=\frac{K_{H}}{K_{L}}$
$Y_{H},Y_{L}(DN)$	Pixel output of the high gain image and low gain image
$K_{H},K_{L}(DN/e^{-})$	Conversion factor for high gain image and low gain image
$O_{H},O_{L}(DN)$	Black level offset of high gain image and low gain image
$T_{H}(DN)$	The threshold value for conversion between low gain image and high gain image
